# Clinical manifestations and perinatal outcomes of pregnant women with COVID-19: a systematic review and meta-analysis

**DOI:** 10.1038/s41598-020-75096-4

**Published:** 2020-10-22

**Authors:** Jeong Yee, Woorim Kim, Ji Min Han, Ha Young Yoon, Nari Lee, Kyung Eun Lee, Hye Sun Gwak

**Affiliations:** 1 College of Pharmacy and Graduate School of Pharmaceutical Sciences, Ewha Womans University, 52 Ewhayeodae-gil, Seodaemun-gu, Seoul, 03760 Korea; 2 College of Pharmacy, Chungbuk National University, 660-1, Yeonje-ri, Osong-eup, Heungdeok-gu, Cheongju-si, 28160 Korea

**Keywords:** Diseases, Health care

## Abstract

This systematic review and meta-analysis aimed to evaluate the impact of COVID-19 on pregnant women. We searched for qualified studies in PubMed, Embase, and Web of Science. The clinical characteristics of pregnant women with COVID-19 and their infants were reported as means and proportions with 95% confidence interval. Eleven studies involving with 9032 pregnant women with COVID-19 and 338 infants were included in the meta-analysis. Pregnant women with COVID-19 have relatively mild symptoms. However, abnormal proportions of laboratory parameters were similar or even increased, compared to general population. Around 30% of pregnant women with COVID-19 experienced preterm delivery, whereas the mean birth weight was 2855.9 g. Fetal death and detection of SARS-CoV-2 were observed in about 2%, whereas neonatal death was found to be 0.4%. In conclusion, the current review will serve as an ideal basis for future considerations in the treatment and management of COVID-19 in pregnant women.

## Introduction

The recent outbreak of the coronavirus disease 2019 (COVID-19) pandemic has called for a prompt response from the scientific community. As of July 30, 2020, confirmed infections have amounted up to over 17 million cases, with casualties reaching an alarming number of over 667,000^1^. The fact that the disease is actively spreading in the world implies that we must prepare for the worst; as a consequence, labs worldwide have pooled their efforts to identify possible therapeutic methods, estimate future progression trends of the pandemic, and sort out the most vulnerable from existing data in order to prepare patient-specific measures.

In this context, many research papers have shed light on the varying effect of COVID-19 depending on patient characteristics, including age and smoking^2–8^. Another important population that deserves meticulous consideration during the COVID-19 pandemic is the pregnant. Pregnancy is a state that is particularly susceptible to infectious diseases, and it is unsurprising that viral infections may affect pregnancy outcomes; previous literature has revealed that viral respiratory illnesses may lead to a higher risk of obstetric complications and adverse perinatal outcomes^9,10^, primarily due to changes in the immune response^11^. Furthermore, previous literature on the impact of the 2009 H1N1 influenza virus or the Zika virus suggest that infectious diseases may increase complications and even exhibit fatal effects on pregnant women^12,13^. Such acknowledgement has led to vigorous investigation from many research groups on the impact of COVID-19 in pregnant patients^14–16^; yet the need to accumulate, organize and analyze such data is evident, given the urgency of the situation.

To that end, this study aims to systematically review previous literature on the impact of COVID-19 on pregnant women. Several important issues, such as perinatal outcome or vertical transmission, are additionally raised in the prognosis of COVID-19 in the pregnant population. Based on ample pre-existing evidence, the current paper attempts to unravel meaningful factors that may aid medical personnel in dealing with such issues and to discover symptoms or phenomena that are specific to the pregnant population.

## Results

A total of 3765 records were identified from searches of three databases, and 1720 duplicates were excluded. After removing 1865 studies during title and abstract screening, 180 were selected for full-text review. Thereafter, 169 articles were excluded for the following reasons: reviews and expert opinions (n = 55); case reports with less than 10 cases (n = 52); irrelevant studies (n = 17); studies without confirmation of COVID-19 by polymerase chain reaction (PCR) tests (n = 9); studies only including the infants born to mothers without confirmed COVID-19 (n = 7); and overlapping studies (n = 29). Hence, eleven studies on 9032 pregnant women with COVID-19 and 338 neonates were ultimately included for meta-analysis^17–27^.

The main characteristics of the included studies are listed in Table 1. All studies were conducted in China. The median age and gestational age at admission of the study population ranged from 28 to 34 years and from 31.3 to 37.3 weeks, respectively. The Cesarean delivery rate varied across studies from 18.2 to 100%. The quality score ranged from 8 to 10.

**Table 1:** Characteristics of studies included in the meta-analysis.

Study ID	Number of pregnant women	Number of neonates	Maternal age^a^ (years)	Gestational age at admission (weeks)	C-section (%)	Setting	Enroll period	Quality score
Dória et al.^17^	12	11	31.9 ± 5.4	37.5 ± 2.9	6 (55.0)	Hospital Pedro Hispano in Portugal	From March 25 to April 15, 2020	10
Ellington et al.^18^	8207	N/A	N/A	N/A	N/A	US CDC data	From January 22 to June 7, 2020	8
Liu et al.^19^	13	10	29.7 ± 4.0	34.0 ± 4.0	10 (100)^b^	China CDC data	From December 8 2019 to February 25 2020	10
Lumbreras-Marquez et al.^20^	308	N/A	N/A	N/A	N/A	Mexican Ministry of Health data	Up to May 17, 2020	9
Mohr-Sasson et al.^21^	11	N/A	28 (24–35)	36.4 (IQR: 35.2–38.5)	2 (18.2)^c^	Sheba Medical Center in Israel	From March to Apr, 2020	10
Nayak et al.^22^	141	134	N/A	N/A	67 (50.0)	Tertiary Care Hospital in India	From April 1 to May 15, 2020	10
Pereira et al.^23^	60	23	34 (22–43)	32 (5–41)	5 (21.7)^d^	Puerta de Hierro University Hospital Madrid, Spain	From March 14 to April 14, 2020	10
Savasi et al.^24^	77	57	32 (15–48)	37.3 (5.3–41.0)	22 (38.6)	12 hospitals in Italy	From February 23 to March 28, 2020	9
Sentilhes et al.^25^	38	17	31.1 ± 6.4	29.3 ± 8.5	7 (41.2)^e^	Strashbourg University Hospital in France	From March 1 to April 3, 2020	10
Vivanti et al.^26^	100	36	33.7 (29–36.7)	31.3 (IQR: 25.6–35.6)	16 (48.0)^f^	4 hospitals in France	From March 12 to April 13, 2020	10
Yan et al.^27^	65	50	30.3 ± 3.7	36.7 (33.8–38.4)	44 (88.0)^g^	25 hospitals within and outside of Hubei province, China	From January 20 to March 24	9

*COVID-19* Coronavirus disease 2019, *NA* not available, *NHC* National Health Commission, *SARS-CoV-2* SARS-coronavirus 2, *WHO* World Health Organization.

^**a**^Data are represented as mean ± SD or median (range).

^b^Fetal distress (in three), premature rupture of the membrane (in one) and stillbirth (in one).

^c^Related to the SARS-CoV-2 infection (in one) and non-reassuring fetal monitor (in one).

^d^Maternal respiratory failure with breech presentation (in one), non-progression of labor (in two), induction failure (in one) and abnormal maternal laboratory levels (in one).

^e^Related to SARS-CoV-2 infection (in six).

^f^Related to SARS-CoV-2 infection (in 13), maternal respiratory distress (in 12) and major coagulopathy (in one).

^g^Related to SARS-CoV-2 infection (in 33), previous cesarean delivery (in 16), fetal distress (in nine), and non-progression of labor (in five).

The meta-analysis results of symptoms of pregnant patients with COVID-19 are shown in Table 2. Among the pregnant patients infected by severe acute respiratory coronavirus 2 (SARS-CoV-2), fatigue was the most prevalent symptoms (54.5%), followed by cough (50.1%) and fever (27.6%). Other common symptoms such as dyspnea, myalgia, and sore throat were observed in about 21%, 16%, and 11% of pregnant women with COVID-19, respectively. The prevalence of diarrhea was less than 10%. In terms of laboratory findings, approximately 48%, 43% and 36% of infected pregnant women had elevated CRP, lymphopenia, and leukocytosis, respectively. The results presented in Table 3 show maternal baseline comorbidities. The prevalence of hypertension (including pregnancy-induced hypertension) and diabetes (including gestational diabetes) was 3.7 and 4.2%, respectively, whereas 4.7% of pregnant women with COVID-19 had asthma.

**Table 2:** Meta-analysis of maternal symptoms.

	Number of studies	Event N	Total N	Prevalence (%)	95% CI	I^2^ (%)
Fever	8	1395	8571	27.6	24.4–71.3	97.8
Cough	7	2018	8560	50.1	26.5–71.7	97.9
Sore throat	4	962	8385	10.5	9.8–11.1	0
Dyspnea	7	1130	8560	20.7	12.5–30.4	88.5
Diarrhea	3	505	8310	6.5	1.6–14.0	77.9
Myalgia	3	1354	8372	16.3	9.5–24.5	80.1
Fatigue	4	60	127	54.5	5.2–98.6	97.1
Lymphopenia	5	108	258	42.6	29.3–56.4	77.1
Leukocytosis	5	41	113	35.6	26.0–45.8	10.8
Elevated CRP	3	86	180	47.7	40.4–55.2	0

*N* number, *CRP* C-reactive protein.

**Table 3:** Meta-analysis of maternal baseline comorbidities.

	Number of studies	Event N	Total N	Prevalence (%)	95% CI	I^2^ (%)
Hypertension^a^	5	24	524	3.7	2.1–5.8	0
Diabetes^b^	6	218	8730	4.2	2.4–6.3	49.1
Asthma	3	18	420	4.7	0.4–12.1	72.7
Chronic respiratory disease	2	411	8515	2.5	0.0–8.3	95.4
Chronic kidney disease	2	14	8515	0.2	0.0–1.0	67.1

*N* number.

^a^Including pregnancy-induced hypertension.

^b^Including gestational diabetes.

The pregnancy and perinatal outcomes of pregnant patients who were infected by SARS-CoV-2 are presented in Table 4. Around 30% of pregnant women with COVID-19 experienced preterm delivery, whereas premature rupture of membranes and fetal distress were observed in about 2%. The mean birth weight was 2855.9 g (95% CI 2634.9–3076.9 g) and the prevalence of small-for-gestational-age births was estimated as 17.4% (95% CI 0–56.0%). Mean Apgar scores at 1 min and 5 min were 8.8 (95% CI 8.6–9.0) and 9.2 (95% CI 8.3–10.1), respectively. Fetal death was observed in about 2%, whereas neonatal death was found to be 0.4%.

**Table 4:** Meta-analysis of pregnancy and perinatal outcome.

	Number of studies	Event N	Total N	Prevalence (%) or mean	95% CI	I^2^ (%)
PROM	3	2	63	2.4	0.0–13.8	50.5
Fetal distress	2	10	63	15.1	6.8–25.6	0.0
Preterm delivery (< 37 week)	6	54	190	28.6	17.8–40.6	61.9
Birthweight (g)	4	N/A	N/A	2855.9	2634.9–3076.9	76.1
Small-for-gestational-age birth	3	9	64	17.4	0–56.0	88.9
Apgar score at 1 min	3	N/A	N/A	8.8	8.6–9.0	26.4
Apgar score at 5 min	3	N/A	N/A	9.2	8.3–10.1	97.9
Fetal death	4	10	63	2.4	0.5–5.4	1.5
Neonatal death	3	1	103	0.4	0.0–3.6	0
SARS-CoV-2 positive	6	5	154	1.8	0.0–5.3	0

*N* number, *PROM* premature rupture of membranes, *SARS-CoV-2* SARS-coronavirus 2, *NA* not available.

In the present study, detection of SARS-CoV-2 was observed in about 2% of the population; a total of five newborns were reported as SARS-CoV-2 positive. Among them, three newborns with vaginal delivery received swab specimen tests on the first day after birth, and one newborn with cesarean delivery was tested on the seventh day. While four SARS-CoV-2 positive newborns were roomed-in and breastfed, data on one neonate was unavailable.

## Discussion

The first notable finding of this study is the difference in common COVID-19 symptoms between pregnant patients and non-pregnant patients. Well-known symptoms of COVID-19 include fever, cough, and dyspnea; in a previous study on non-pregnant COVID-19 patients, the proportion of those who show each symptom was shown to be 83%, 82%, and 31%, respectively^28^. In our study of pregnant women, the proportions decreased to 28%, 51%, and 21%, indicating relatively mild symptoms. This result was in line with another previous study by Liu et al*.* that compared pregnant and non-pregnant COVID-19 patients, where more pregnant patients were classified as mild or common^29^.

Milder symptoms in pregnant COVID-19 patients may be explained by the younger average age compared to the general COVID-19 patient population; additionally, as there was much fewer comorbidities, symptoms might have appeared to be less profound in the pregnant population. In fact, chronic diseases such as hypertension and diabetes were less observed in our study than in previous studies not restricted to pregnant women; the prevalence of hypertension, diabetes and chronic kidney disease were found to be 3.7%, 4.2% and 0.2% in our study, whereas those in a study with 5700 patients with COVID-19 were 56.6%, 33.8% and 5.0%, respectively^30^.

Unlike common symptoms, various abnormalities of laboratory parameters showed a similar or even increased trend in pregnant women with COVID-19 compared to general patients. A previous meta-analysis using patients with COVID-19 reported that proportions of leukocytosis, lymphopenia, and elevated CRP levels were 17%, 43%, and 58%, respectively^31^, while those in our study were 36%, 43%, and 48%. This gap between the pregnant and general patient population was probably attributable to changes in the immune response in pregnancy^11^.

Pregnancy is regarded as an immunocompromised status in some aspects, especially since maternal immunity is altered to tolerate fetal antigens by suppressing cell-mediated immunity^32^. However, the number of immune cells, such as macrophages, natural killer (NK) cells and regulatory T cells, can increase even in normal pregnancy^33^. Accordingly, pregnancy yields a unique immunity status^32^ and may experience increased susceptibility to certain intracellular pathogens, including bacteria and viruses^34^. Furthermore, it has been reported that the number of leukocytes (mostly accounting for neutrophils) increased early in gestation and remained elevated, which can be explained by the increased corticosteroid and estrogen levels^35^.

Of utmost importance is the effect of COVID-19 infection in pregnant women; in this regard, pregnancy outcomes were observed in the current study. In total, 29% of the study sample exhibited preterm delivery, a strikingly high number compared to the norm, which was reported to be between 5 and 18%^36^. In previous research on pregnant patients in past *coronaviridae* outbreaks, namely severe acute respiratory syndrome (SARS) and Middle East respiratory syndrome (MERS), the proportions of pregnant patients that experienced preterm delivery were 29% and 32%, respectively, which were approximately similar to the ratio in pregnant COVID-19 patients^37^.

In addition, data on neonates born from COVID-19 patients showed varying tendencies compared to the non-infected. Average Apgar scores at 1 min and 5 min were recorded to be an adequate 8.8 and 9.2, respectively, while average body weight was 2855.9, which is considered normal. On the other hand, proportions of fetal distress was 15.1%, displaying dissimilarity to those of non-inflicted cases, which were 6.8%^38,39^. While the rate of fetal death in China has been reported to be 0.43%, the present study population exhibited a rate of 2.4%, showing a significant gap as well^40^. Finally, there was one case (0.4%) of neonatal death in our study, which indicated that the presence of COVID-19 in the mother did not seem to result in a higher probability of neonatal death.

Vertical transmission is another crucial issue, primarily as newborns possess an underdeveloped protective system against external sources of potential harm. Yet controversy had existed regarding whether SARS-CoV-2 can be transmitted from the mother to the fetus within the uterus. Research on previous coronavirus outbreaks fail to provide definite evidence for or against vertical transmission in pregnant patients; on the other hand, in other respiratory viruses such as influenza or respiratory syncytial virus (RSV), cases of vertical transmission have been reported^41,42^. In the present study, a total of five newborns were reported as SARS-CoV-2 positive, suggesting that vertical transmission of COVID-19 may not be negligible.

In addition, a case report showed that virus-specific antibodies (IgG in 5 and IgM in 2 among 6 neonates) were detected in serum samples of the neonates born to pregnant COVID-19 patients, although SARS-CoV-2 itself was undetected by PCR tests^43^. Detection of IgM was of interest because unlike IgG, which can be transferred passively across the placenta from the mother to fetus^44^, IgM is known to be too large to cross the placenta^45^ and might be produced by the fetus itself; accordingly, positive IgM in neonates might indicate past exposure, implying the possibility of vertical transmission of SARS-CoV-2. However, in a study of Ben-Hur et al., transplacental passage of IgM was detected in cases of severe inflammation^46^. Detection of IgM in neonates should be interpreted with caution regarding whether the IgM in neonates was produced by fetus after vertical transmission or transferred from the mother.

The present meta-analysis bears a few limitations that should be considered in the interpretation of results. Detailed clinical manifestations were not available in the selected studies. In addition, the associations between clinical features and outcomes were not provided. Nevertheless, as a systematic review and meta-analysis on pregnant COVID-19 patients, the current review will serve as an ideal basis for future considerations in the treatment and management of COVID-19 in pregnant women.

## Methods

### Literature search strategy

**Figure 1:**
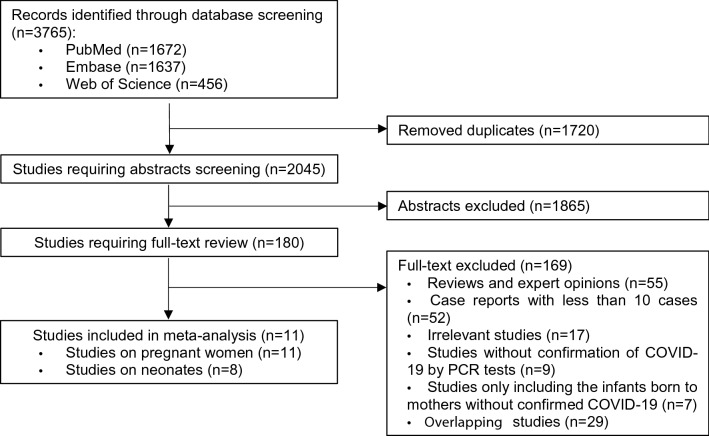
Flow diagram of study selection.

Two researchers separately searched PubMed, Embase, and Web of Science for studies on clinical characteristics of pregnant women with COVID-19 and their neonates, published between 1 January 2020 and 20 July 2020. The following search terms were used [(Pregnan * OR gestation * OR maternal OR fetal OR perinatal OR obstetric * OR neonate * OR infant * OR newborn*)] AND [(“coronavirus disease 2019” OR “coronavirus disease-19” OR “COVID-19” OR “2019-nCoV” OR “SARS-CoV-2” OR “novel coronavirus”)]. There was no restriction on language of publication. Duplicates and obviously irrelevant studies were excluded through initial screening of titles and abstracts, and the remaining articles were further reviewed according to inclusion and exclusion criteria. A flow diagram summarizing the study selection process is shown in Fig. 1.

### Inclusion and exclusion criteria

The following criteria were used to identify eligible studies: (1) describing the clinical characteristics of pregnant women with COVID-19 and their neonates; (2) in which COVID-19 diagnosis was confirmed by PCR tests; and (3) using an observational study design. Exclusion criteria were: (1) reviews and expert opinions; (2) in vitro or animal studies; (3) studies on infants born to mothers without confirmed COVID-19 infection; or (4) case series or case report with less than 10 cases. In instances of overlapping data, only the most recent and comprehensive data were included in the meta-analysis.

### Study selection, data extraction and quality assessment

Two investigators separately selected publications and extracted data, and discrepancies were resolved by consensus. The following information was extracted from each study: name of the first author, publication year, study setting, study design, patient age, gestational age at admission, percentage of Caesarean section, and diagnosis criteria of COVID-19. Also, prevalence of clinical symptoms, comorbidities, and maternal and fetal outcomes were extracted, along with mean and standard deviation of birth weight and Apgar scores. In the case of outcomes with lack of data, we utilized and additionally analyzed studies that had been excluded due to overlap; for example, the meta-analysis of leukocytosis was performed using additional studies^47–51^, as the included studies did not have data for leukocytosis. The methodologic quality of the selected studies was evaluated using the Joanna Briggs Institute critical appraisal checklist for case series by two researchers, independently^52^. This checklist contains 10 questions for assessing the risk of bias in the design and conduct of the study, and the higher the scores, the higher the study quality.

### Statistical analysis

The clinical characteristics of pregnant women with COVID-19 and their neonates were reported as means and proportions with 95% confidence interval (CI). Statistical heterogeneity was assessed using I^2^ statistics. To avoid bias from studies with a zero-event rate, proportions were transformed via the Freeman–Tukey Double ArcSine method. A random-effects model (DerSimonian-Laird method) was applied to consider the heterogeneity within and between studies and to give a more conservative estimate of statistical confidence. All statistical analyses were performed using R software (version 3.6.0) with meta package^53^. The review was written based on Preferred Reporting Items for Systematic Reviews and Meta-analyses (PRISMA) guidelines.
